# Strategies of preventing ureteral iatrogenic injuries in obstetrics-gynecology

**Published:** 2012-09-25

**Authors:** M Cirstoiu, O Munteanu

**Affiliations:** Clinic of Obstetrics and Gynecology, University Emergency Hospital, Bucharest

**Keywords:** iatrogenic ureteral lesion, prevention, risk factors

## Abstract

The incidence of ureteral lesions varies between 0.1% and 30% depending on the type of the surgical intervention. However, the surgical interventions in Obstetrics and Gynecology are responsible for 50% of the total iatrogenic ureteral lesions. Sadly, only 1/3 of the iatrogenic ureteral lesions are recognized during surgeries and 25% of the unrecognized cases of ureteral lesions lead towards the loss of the damaged kidney, while a delayed diagnostic may also lead to a progressive deterioration of the renal function. On this matter, of decreasing the rate of morbidity and the following forensic risks, the gynecologist surgeon must be able to anticipate the potential apparition of a specific ureteral lesion, based on the known risk factors of the patient, so that he can then prevent the iatrogenic ureteral lesion.

## The incidence of iatrogenic ureteral injuries

The incidence of ureteral injuries varies between 0,1% and 30%, according to the type of the surgery. However, in Obstetrics-Gynecology, surgery is responsible for 50% of the total of iatrogenic urethral injuries; they occur with a frequency of 1% in the case of abdominal hysterectomy, and of 0,1% in case of vaginal hysterectomy [**4,6,7**]. In laparoscopic surgery, the incidence is unknown for the time being, but it is considered bigger than in traditional surgery. Although the prevalence of the infringement of the uterus in cancer surgery is high, the majority of the iatrogenic ureteral injuries occur during surgery for benign tumors. (for example, 38% occur in the case of treatment for endometriosis) [**4**]. 

## Risk factors for an iatrogenic ureteral injury 

• Anatomical

• Anatomo-pathological

• Technical

Risk factors determined by the normal anatomy of the ureter:

- the ureter is a retro and subperitoneal organ but it also remains adherent to the posterior parietal peritoneum together with which it moves when this one is mobilized and thus it can be mistaken for a fold of the peritoneal and cut [**1,3,5**]. 

- the ureter has an intimate relation with high caliber elements which pump blood into the uterus and the annexes - the lombo-ovarian ligament (at the level of the upper strait basin) and the uterine artery in the broad ligament of the uterus, and thus, it can be ligature-clipped and cut together with them; the ureter can also be angulated, with the appearance of a secondary obstruction, if the ligature of ovarian or uterine artery is made very close to the ureter [**1,2,3,5**]. 

- the ureter has variable trajectory-especially in the parietal segment of the pelvic area [**1,3**]. 

 - the ureter is poorly vascularized–the terminal branches of the arteries, veins and lymphatic ureteral get anastomized and form a vascular periureteral network – which explains the avascular necrosis which appears a couple of days after the surgery, after stripping, as well as its destruction during the extensive dissections of the ureter [**1,3,5**]. 

The Anatomo-pathological risk factors that determine the modification of the pelvis anatomy are the following [**4,6,7,8**]:

- Congenital anomalies of the ureter or of the kidney 

- Position changes of the ureter through: 

• The dimension of the uterus > 12 weeks

• Prolapse 

• Bulky tumors 

• Tumors of the large ligaments

- Adherent 

• Post surgery

• Endometriosis

• BIP

• Neoadjuvant radiation 

Technical risk factors 

Massive intraoperative bleeding which forces “blind” clamping for the haemostasis 

- Bladder coexistent injury which masks the ureteral injury 

- Technical difficulties appear either due to bulky tumors or with the invasion in adjacent structures, which modifies the pelvis anatomy, or to the surgeon’s lack of experience. 

Preventive strategies to reduce the risk of ureteral injury:

- General preventive strategies

- Specific preventive strategies

General preventive strategies 

Preoperative measures:

All the patients who are going to undergo a pelvis surgery need a preoperative pelvic ultrasound that must exclude or warn the surgeon that there is an anatomical variant or aberrant trajectory of the ureter. In selected cases, according to the following: the history of the illness (i.e. endometriosis), family history (i.e. congenital anomalies of the ureter and kidney in Ist degree relatives), physiological persons (i.e. – at multiparous because of the relaxation of the means for ovary fixing, there is a change of relations between the ureter and the ovary – the retroligamentary segment, during the ovary mobilization, standing for one of the most frequent localizations of the iatrogenic ureteral injuries) or personal pathological history (i.e. – multiple pelvis surgery) the following can be used: intravenous urography, CT abdomen and pelvis with contrast material, RMN abdomen and pelvis or retrograde urography [**4,6,7,8**].

Intraoperative measures:

- The proper surgical approach 

- The proper exposure of structures 

- The avoidance of blind clamping of the blood vessels 

- The dissection with routinely highlight of the ureter and direct visualization 

- The careful mobilization of the bladder 

- The cautious use of the electrocautery 

Specific preventive strategies:

According to the type of the surgery and the way of dealing with it, some gestures and steps should be taken into account in order to prevent iatrogenic ureteral injuries. No matter the way of dealing is used, the ureter should be identified in any type of pelvis surgery, and, in the difficult ones, its highlight should be accomplished from a retroperitoneal point of view [**4,6**].

**Fig. 1:**
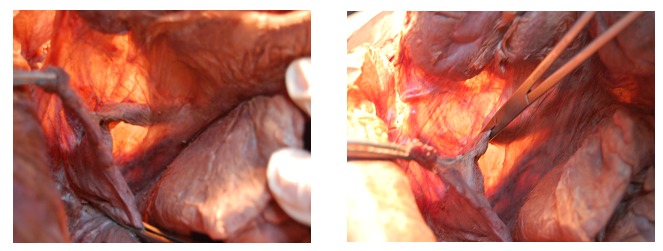
Clamping the lombo-ovarian ligament as close to the uterus as possible to avoid the lesion of the uterus at the level of the superior strait – evidenced in a cadaver by transillumination

During abdominal surgery:

- The use of intracapsular techniques, with the ligature and sectioning of cardinal ligaments, uterosacrate and vezicouterin as close as possible to the uterus

- The ligature and sectioning of the uterine vessels as close as possible to the uterus because the uterine vessels in the subligamentar segment of the pelvine area is the most frequent place in which the ureter is injured during abdominal hysterectomy abdominal (either angulated, either clipped, ligaturated and cut once with the uterine vessels especially during extracapsular techniques) [**4,6,7,8**]

- The ligature and the sectioning of the lombo-ovarian ligament as close as possible to the ovary, because, at the upper strait basin, the ureter can be clipped, ligaturated and cut along with it (as both elements going towards the interior get far from one another) (**Fig. 1**) [**4,8**]

During vaginal surgery [**4,6,7**]: 

- The ligature and sectioning of the uterine arteries as close as possible to the uterus

- The proper exposure of the vesico-uterine area through the pulling of the cervix towards the lower, in order to avoid the injury of the ureter in the preligamentar segment of the pelvine area during the cutting of the vesico-uterine ligaments vezico-uterine during vaginal hysterectomy. 

- During the anterior colporaphy: 

• avoiding the dissection in lateral 

• avoiding the profound sutures.

During laparoscopic surgery [**4,7**]:

- The mobilization of the fallopian tube far from the pelvic sidewalls before the electrocoagulation, in order not to injure the ureter in the preligamentar segment of the pelvine area. 

- the insurance of the haemostasis at the level of the uterosacrate ligaments with sutures or clips, instead of the electrocoagulation, because, at this level, the ureter is most frequently injured, during laparoscopic hysterectomy. 

**Fig. 2:**
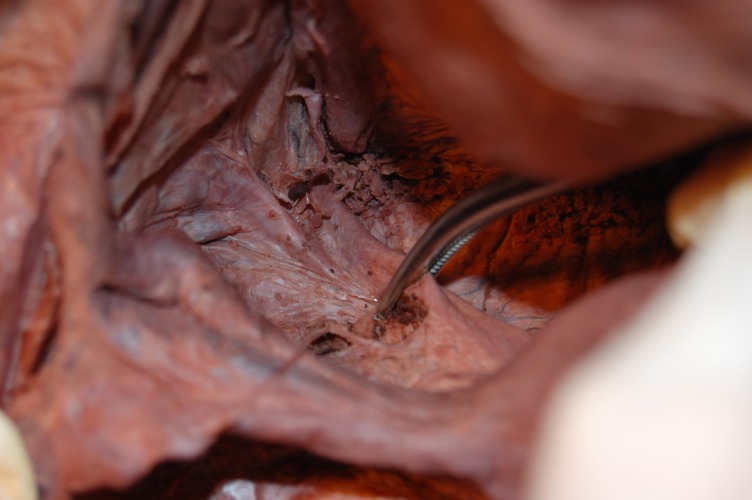
Clamping the uterosacrate ligament – evidenced in a cadaver

- During hysterectomy, in order to insure the haemostasis on the trak? and at the level of cardinal ligaments, the electrocoagulation should be avoided, because there can occur ureteral injuries through ischemia, due to the destruction of the periureteral vascular network. 

## Conclusions

It is most likely that ureteral injuries occur during difficult surgeries. However, most studies demonstrated that iatrogenic ureteral injuries occur during routine surgery. This apparent paradox is due to a false feeling of security that the surgeon develops in time. But, for the decrease of morbidity (only 1/3 from the ureteral iatrogenic injuries are recognized intraoperatory and 25% from the unknown cases lead to the loss of the affected kidney, the diagnosis delay leading to the progressive deterioration of the renal function [**4,7**] and of the subsequent medico legal risks, the surgeon should be able to foresee the potential developing of a specific ureteral injury, based on the known risk factors of the patient, and thus, he can be able to prevent the iatrogenic ureteral injury. 

## References

[1] Filipoiu  FM (2005). Anatomia omului. Aparatul urinar. Spatiul retroperitonea.

[2] Lupu  G (2005). Anatomia omului. Aparatul genital.

[3] Moore  KL (2006). Clinically Oriented Anatomy – fifth edition.

[4] Murad  AWA (2008). Ureteral Injuries In Obstetrics And Gynecology.

[5] Schenkman  S (2011). Medscape reference.

[6] Thompson  JD (1997). Operative injuries to the ureter: prevention, recognition, and management - Te Linde's Operative Gynecology 8th edition.

[7] Vasavada  SP (2011). Ureteral Injury During Gynecologic Surgery. Medscape reference.

[8] Zanoschi C (2005). Anatomia ureterului pelvin la femeie. Jurnalul de Chirurgie.

